# Phase recognition in manual Small-Incision cataract surgery with MS-TCN + + on the novel SICS-105 dataset

**DOI:** 10.1038/s41598-025-00303-z

**Published:** 2025-05-21

**Authors:** Simon Mueller, Bhuvan Sachdeva, Singri Niharika Prasad, Raphael Lechtenboehmer, Frank G Holz, Robert P Finger, Kaushik Murali, Mohit Jain, Maximilian W M Wintergerst, Thomas Schultz

**Affiliations:** 1 University Hospital Bonn, Department of Ophthalmology, Bonn, Germany; 2 Microsoft Research, Bengaluru, India; 3 Sankara Eye Hospital, Bengaluru, Karnataka India; 4 Department of Ophthalmology, University Medical Center Mannheim, Heidelberg University, Mannheim, Germany; 5 Augenzentrum Grischun, Chur, Switzerland; 6 Department of Computer Science, B-IT, University of Bonn, Bonn, Germany; 7 Lamarr Institute for Machine Learning and Artificial Intelligence, Bonn, Germany; 8 Dept. of Ophthalmology, University Hospital Bonn, Venusberg-Campus 1, 53127 Bonn, Germany

**Keywords:** Small-Incision Cataract Surgery, Phacoemulsification, Artificial Intelligence, Temporal Action Segmentation, Phase Recognition, Video Dataset, Translational research, Lens diseases, Computer science, Scientific data

## Abstract

Manual Small-Incision Cataract Surgery (SICS) is a prevalent technique in low- and middle-income countries (LMICs) but understudied with respect to computer assisted surgery. This prospective cross-sectional study introduces the first SICS video dataset, evaluates effectiveness of phase recognition through deep learning (DL) using the MS-TCN + + architecture, and compares its results with the well-studied phacoemulsification procedure using the Cataract-101 public dataset. Our novel SICS-105 dataset involved 105 patients recruited at Sankara Eye Hospital in India. Performance is evaluated with frame-wise accuracy, edit distance, F1-score, Precision-Recall AUC, sensitivity, and specificity. The MS-TCN + + architecture performs better on the Cataract-101 dataset, with an accuracy of 89.97% [CI 86.69–93.46%] compared to 85.56% [80.63–92.09%] on the SICS-105 dataset (ROC AUC 99.10% [98.34–99.51%] vs. 98.22% [97.16–99.26%]). The accuracy distribution and confidence-intervals overlap and the ROC AUC values range 46.20 to 94.18%. Even though DL is found to be effective for phase recognition in SICS, the larger number of phases and longer duration makes it more challenging compared to phacoemulsification. To support further developments, we make our dataset open access. This research marks a crucial step towards improving postoperative analysis and training for SICS.

**Supplementary Information:**

The online version contains supplementary material available at 10.1038/s41598-025-00303-z.

## Introduction

Cataract is the word-wide leading cause for blindness, and there is a worldwide inequity in cataract burden, as blindness due to cataract is approximately 10 times more frequent in low- and middle-income countries (LMICs)^1,2^. However, surgical outcomes for cataract surgery in LMICs are often poor due to limitations in infrastructure, training and management of complications^2–9^. Therefore, there is a great necessity for improving outcomes of cataract surgery in LMICs^9–16^. One possible solution is video recording of surgeries which allows for self-evaluation, video-based coaching and monitoring of results. It has been shown that implementation of these approaches is directly associated with improvement of surgical results^9,11,17^. However, these steps are expensive in time- and personnel-cost and therefore usually not available in LMICs^9,17^. Automated video annotation would alleviate the personnel/time requirement and reduce cost. However, automated analysis for cataract surgery has only been done for phacoemulsification cataract surgery^18^, which is largely not available in low-resources settings. The mainly used, most appropriate, and most cost-effective cataract surgical technique for low-resources settings is small incision cataract surgery (SICS)^2,19–28^. Yet, so far, no automated video analysis has been done for SICS.

Automated annotation of cataract surgical videos can be achieved through recent advancements in artificial intelligence (AI), particularly in deep learning (DL). DL algorithms make it possible to generate insights from data without manual feature crafting as was done in the past^29^. The integration of AI into surgery analysis could improve surgical outcomes and enhance efficiency for healthcare practitioners. Important steps for surgical evaluation are the identification of critical phases, tracking of instruments and complication recognition^18,30,31^. As a long-term goal, grading of SICS quality could be automated by AI with standardized assessment rubrics like Sim-OSSCAR^32^.

Therefore, there is an interest in generating open-access data for SICS and testing existing algorithms on it. In a collaboration between the University Hospital Bonn, the University of Bonn, Sankara Eye Foundation India, and Microsoft Research India, we aim to create such a dataset and develop DL algorithms for SICS in the three domains identified in our review^18^.

In this work, we provide the first public dataset for SICS, the “SICS-105 dataset”, from 105 patients (without complications) annotated by four ophthalmologists from the Sankara Eye Hospital in Bangalore, India (see Sect. Materials and methods). This data provides a strong foundation for postoperative video analysis and phase segmentation. Predicted phases could be used in the clinic to recognize surgical protocol derivations, identify phases with a higher risk for complications and be the basis for quality assessment. Currently, there is no established threshold of predictive performance for phase segmentation in the literature, therefore we adopted a state-of-the-art DL architecture for general temporal segmentation (MS-TCN++) and trained it on the public Cataract-101 phacoemulsification dataset for a performance baseline (Sect. Results). Then we compared these results first with other authors working on phacoemulsification and with the same network retrained on our SICS-105 dataset. We expect to be able to achieve similar predictive performance thus paving the first step for automatic evaluation of SICS for training and - in the long term - improving surgical outcomes.

## Materials and methods

Following, we introduce our data, the used DL architecture, and validation strategy.

### Overview cataract surgeries

A cataract is a clouding of the clear lens of the eye, which lies posterior to the iris and anterior to the vitreous body and retina. This leads to decreased visual acuity, with a common cause being age-related degeneration^2,20,33,34^. Cataract is treated through minimally invasive surgery, such as phacoemulsification and SICS.

#### Phacoemulsification

Introduced in 1967 by Charles Kelaman, this technique utilizes ultrasound to emulsify and remove cataracts. Important steps are described in the literature and visualized in the Cataract-101 publication^35–37^.

**Fig. 1:**
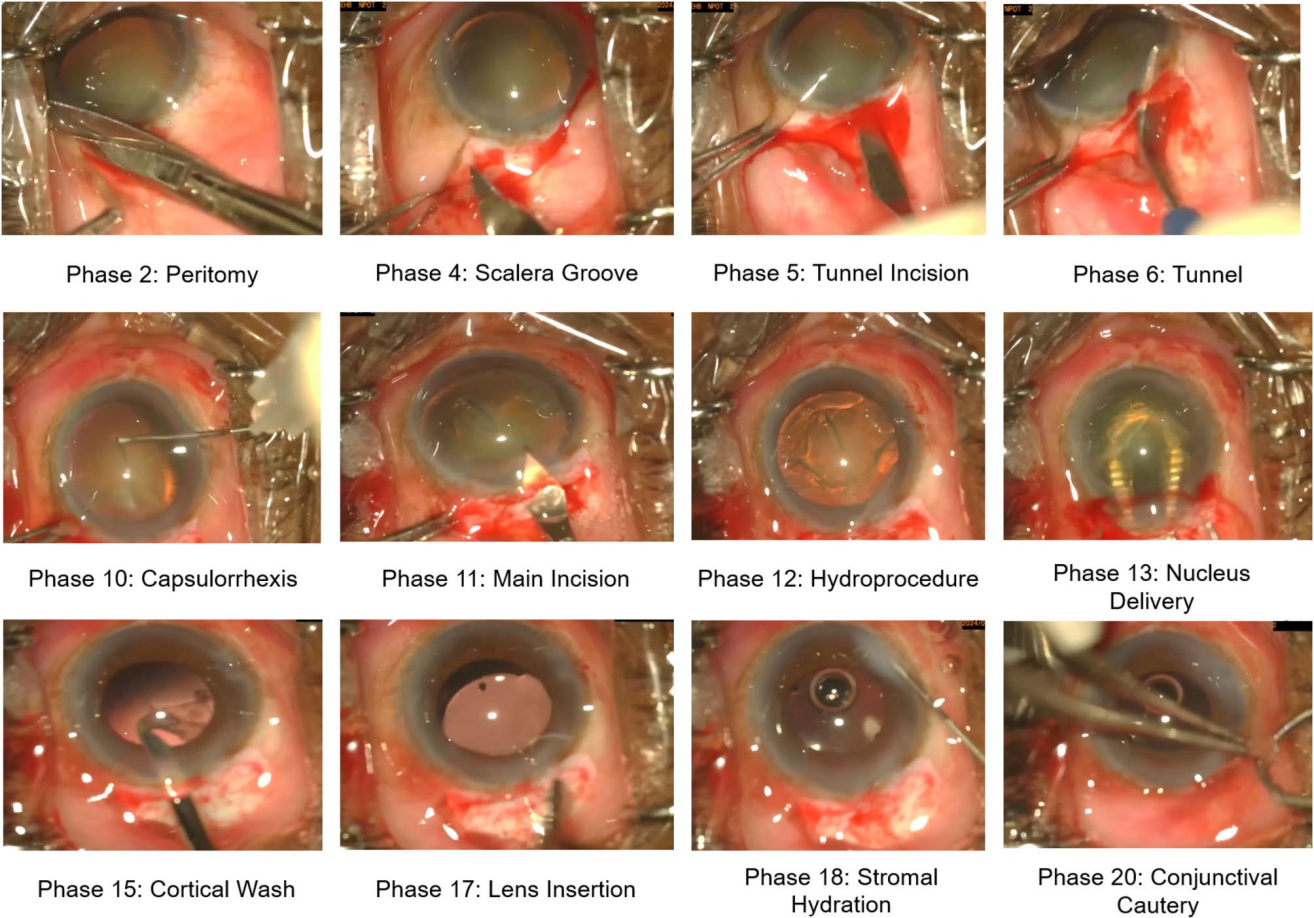
Selection of the most important steps in the novel Small-Incision Cataract Surgery (SICS) dataset. For compactness the following phases are not displayed: bridle suture, cautery, antibiotic injection, OVD injection, tunnel suture, OVD wash, nucleus prolapse, and sideport.

#### Manual Small-Incision cataract surgery

Manual small-incision cataract surgery (from hereon SICS) is frequently used in LMICs due to comparable outcomes to phacoemulsification, cost-effectiveness, and fewer complications^22,23,25,28,38^. The surgeon constructs a tunnel in the sclerocorneal region, injects an OVD to maintain the corneal dome, performs a capsulorhexis, hydroprocedures separate lens layers, and the nucleus is prolapsed and extracted^39^. The remaining lens cortex is removed, an IOL is placed into the capsular bag and the tunnel is closed. Important steps are shown in Fig. 1.

### Dataset details

#### Cataract-101: 

The Cataract-101 dataset is the largest publicly available phacoemulsification collection, comprising 101 surgeries performed by four surgeons at Klinikum Klagenfurt, Austria over 9 months in 2017, with annotations for 10 surgical phases. The total video duration is 14 h and 2 min, at 720 × 540 pixels and a framerate of 29 fps. An average recording is 8 min and 20 s long with a standard deviation (σ) of 3 min, 13 s^36^.

**Novel SICS-105**: Our novel SICS dataset consists of 105 recordings performed by surgeons at the Sankara Eye Hospital in Bangalore, India, and annotated by four ophthalmologists over 6 months in 2023-24 with 20 phases. The total duration of the videos is 22 h and 39 min with a resolution of 1920 × 1080 pixels (later downsampled to 960 × 540 pixels) and 30 fps. The average video length is 12 min and 57 s (σ = 4:31 min).

The dataset can be publicly accessed under the following url: 10.5281/zenodo.13847781. The overall study protocol, the data collection and dataset publication were approved by the Institutional Ethics Committee of Sankara Eye Hospital on the 11th of January 2024 in Bangalore, India. The approval number is SHE/BLR/EC/2024/114. All experiments were performed on anonymized data and were performed in accordance with relevant guidelines and regulations. As detailed below informed consent was obtained from all participants before surgery and data collection.

*Patient selection and recruitment*: Patients with impaired best-corrected visual acuity due to cataract (in a clinical investigation from an ophthalmologist) and an indication for SICS, were prospectively recruited at Sankara Eye Hospital Bangalore, India in associated screening camps. The surgery was performed using a digital microscope with a field-of-view recording system. After signing the informed consent, patient data is acquired, anonymized and randomized into training, validation and test sets. Patients without consent form or when their recordings video quality is unusable as determined by an ophthalmologist are excluded.

*Outlier Removal and Processing*: Videos are manually reviewed and recordings showing complications requiring extra phases not encoded in the 20 phases of SICS (e.g. vitrectomy) and 9 videos longer than the mean plus two times the standard deviation were removed (duration > 21:30 min). Videos are scaled down to 720 × 540 pixels using ffmpeg’s lanczos-resampling for better comparability with the Cataract-101 dataset and to counteract blurriness caused by compression by the recording equipment.

Details about phases in both datasets and which phases were fused in SICS-105 are supplied in the supplement (Sect. 8.2).

### Proposed architecture (MS-TCN++)

Action segmentation networks used in the video analysis community can be adapted for surgical phase recognition by retraining on surgical video datasets.

**Fig. 2:**
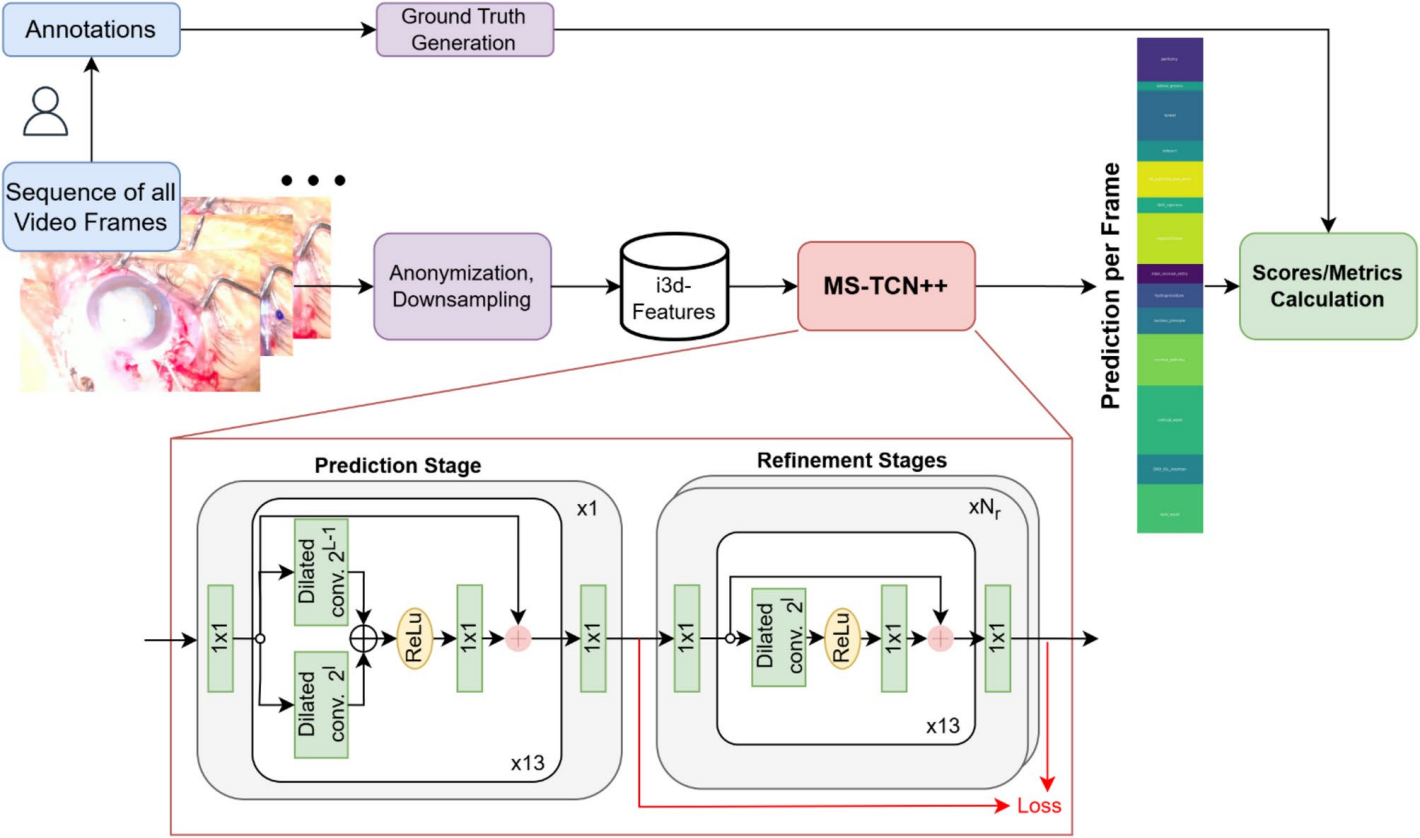
This flowchart displays how data moves through our pipeline for phase recognition in SICS. First, the 105 videos of our collection are manually annotated by ophthalmologists and the ground truth is generated from this information. The raw videos are processed into I3D features, which serve as input for the MS-TCN + + architecture. The red box provides further details on MS-TCN++, adapted from the original publication^40^. The model first generates an initial coarse prediction in the *Prediction Stage* which is subsequently refined through N_r_ *Refinement Stages*. Each stage in our setup consists of 13 layers and utilizes dilated convolutions to integrate an increasing temporal context. The phases predictions output by the Network are then compared with ground truth to calculate various performance metrics (e.g. accuracy).

In this work, we utilized the Multi-Stage Temporal Convolutional Network (MS-TCN++) model due to the publicly available code, the computational light-weight architecture, and promising results. Implementation details can be found in the original publication^40^, but we want to highlight some important cornerstones of the architecture and describe our data pipeline:

Figure 2 illustrates the generation of ground truth annotations and the subsequent video processing pipeline using an example from the SICS-105 dataset. Following annotation by ophthalmologists, I3D features are extracted from the videos and used as input for the MS-TCN + + architecture to predict surgical phases. Performance metrics are calculated by comparing the prediction of the model and the ground truth (for the Cataract-101 data a similar workflow is utilized, but the ground truth is already available from the publishers).

The MS-TCN + + model consists of one *Prediction Stage* and a few *Refinement Stages*, each containing multiple layers. The first stage uses *dual-dilated convolutions* with the intuition that each layer combines information from a local temporal context and more distant timesteps (high receptive field) to generate an initial coarse phase prediction. With an increasing layer index, this narrow local context widens to include more information. After the first stage, the subsequent parameter-sharing *Refinement Stages* incrementally improve the prediction through a simpler module utilizing *single-dilated convolutions* (see Fig. 2, red box).

After each stage, a loss is calculated, and losses are accumulated over all stages for training. The loss combines a cross-entropy loss and a mean-squared-error (MSE) loss for punishing wrong predictions and discouraging over-segmentation. These two losses are balanced by hyperparameter lambda λ.

From our dataset 15% of the surgery videos are set aside as a test set, the remaining videos are randomized into seven folds for training and validation of the MS-TCN + + model. The test set is necessary to avoid overestimation of the model’s performance after hyperparameter tuning and evaluating model modifications. Choosing k = 7 ensures that enough data remains for training (unlike much lower k) while avoiding excessive computational effort, which would result from much higher k^41^. Mathematical i3d features are extracted at a framerate of 15 fps using the extraction library v-iashin/video_features^42^. The groups are used for below described experiments and hyperparameter tuning. For comparison of predictive performance of the network on both datasets, we use the respective test sets.

### Statistical analysis

The Python programming language (version 3.11), the Scikit statistics and the Matplotlib library were used to randomize acquired videos, evaluate results and generate plots.

For quantitative evaluation, we utilize frame-level accuracy, macro-averaged F1 scores, sensitivity and specificity, the area under the receiver operator curve (ROC-AUC) and the precision-recall curve (PR-AUC), and the phase-level edit score (Levenshtein distance)^43^. 95% confidence-intervals (CIs) are calculated with test-set bootstrapping (500 resamplings) for each above-described metric in the test-set comparison^44^.

Further we plotted Confusion Matrices, ROC and PR curves to get a visual estimate of model performance. To investigate the impact of video length on prediction performance, the spearman correlation-coefficient with associated p-value is calculated.

### Ablation studies

For the Cataract-101 dataset we search for the optimal weighting parameter λ between the loss functions, and test alternative losses.

Hyperparameters are tuned through 7-fold cross-validation on the training data (individually for each dataset), with a randomized hyperparameter search-strategy; further details are provided in the source code. After training with optimal parameters, performance on an independent test set is compared with published results from other authors.

With the SICS-105 data, we combined some of the 20 into 13 phases according to advice from two senior ophthalmologists using a confusion matrix analysis (see Supplement Fig. 1), removed un-annotated timestamps between phases and weighted the loss function by class frequency^45^. The aim is to make the dataset comparable to the baseline Cataract-101 data.

## Results

In the following we present results for the public Cataract-101 (used as a baseline) and the novel SICS-105 dataset.

### Architecture optimization and validation on cataract surgery

The original MS-TCN + + publication uses two loss functions for training of the network: a cross-entropy (CE) classification loss and a truncated mean-squared-error (MSE) loss for punishing over-segmentation. These losses are combined through addition and weighting of the MSE loss with the hyperparameter λ.

**Table 1:** Impact of the weighting factor lambda between the cross-entropy classification loss and the smoothing mean-squared-error loss on the neural network, evaluation was done on the validation subset of the data.

Metric	λ = 0.15	λ = 0.20	λ = 0.25	λ = 0.30	λ = 0.35	λ = 0.40	λ = 0.50
Accuracy	83.94	81.50	82.98	87.76	89.83	88.27	86.46
Edit	77.56	76.03	73.67	80.54	86.81	82.12	78.65
ROC AUC	97.37	96.64	97.15	98.60	98.82	98.53	98.33
PR AUC	82.41	78.60	80.73	86.91	91.58	87.34	84.63

Table 1 shows that a lambda value of 0.35 provides the best accuracy of 89.83% and the best ROC-AUC value of 98.82%. This value will therefore be used for all future evaluations.

Replacing the CE loss with a focal loss as proposed by Jiang et al. (accuracy of 88.27 to 88.16%) or adding a dice-loss (accuracy of 88.27 to 89.51%) did only marginally improve performance of our network^46^. Also, enhancing the currently used frame-based i3D features with optical flow data increased the preprocessing time by a factor of 3 and hurt predictive performance with an accuracy of 80.40 versus 83.35% and edit-score of 69.12 versus 75.04%.

#### Hyperparameter optimization

Utilizing the random search optimization strategy, yielded the following optimal hyperparameters (from 50 random combinations).

- Number of layers in the prediction generation stage = 13.
- Number of layers in the refinement stages = 13.
- Number of refinement stages = 4.

#### Comparison with models from the literature

 We applied our optimized MS-TCN + + model to the hold-out testset and compared the results to solutions from other authors for the Cataract-101 data and achieved competitive results across multiple metrics as displayed in Table 2^47–50^. We performed a similar comparison with the IEEE CATARACTS dataset (see Supplement Table 1).

**Table 2:** Comparing our baselines results on the cataract-101 dataset with phase recognition approaches by other authors in the last 10 years. #P = number of phases used for segmentation. NR = Not reported by the author. Best result in bold font, second best is underlined.

Author	#*P*	Accuracy	ROC AUC	PR AUC	Sensitivity	Specificity
**MS-TCN++ (ours) on Cataract-101**	10	0.913	0.994	0.865	0.912	0.978
**Touma**,** 2022**^47^	10	0.959	NR	0.855	0.610	0.962
**Nespolo**,** 2022**^48^	4	NR	0.961	0.960	NR	NR
**Mahmoud**,** 2023**^49^	10	0.990	0.940	NR	0.890	NR
**Fang**,** 2022**^50^	10	0.965	NR	NR	0.952	NR

### Experiments on SICS-105

After addressing differences between the raw SICS-105 dataset and the Cataract-101 dataset as described in Sect. 2, experiments demonstrate that they improve predictive performance. The changes improve predictive performance of the model on the SICS-105 data from an initial low accuracy of 60.42 to 73.01% and then finally to 80.71% (other metrics like edit-distances also improve from 44.79 to 74.87%). A combined class-frequency and sample-frequency weight is used as suggested by Tóth et al. but does not improve performance (accuracy increases from 77.49 to 78.50% but edit distance decreases from 78.86 to 76.17%)^45^.

### Comparison of the results on main datasets

After optimizing the hyperparameters, it is possible to compare results from both video collections as reported in Table 3. We held back a test set of videos (n_Cataract−101_ = 15, n_SICS−105_ = 17) that were not yet seen by the algorithm nor used for their respective optimization.

**Table 3:** Performance evaluation of the MS-TCN + + network on the cataract and SICS datasets with optimized hyperparameters. 95% confidence intervals are reported in brackets (lower, higher). These results were calculated on the test set which was not used for hyperparameter tuning or the earlier described experiments. #P indicates the number of phases in the dataset.

Metric	Cataract-101, #*P* = 10 (95% CIs) in %	SICS-105, #*P* = 13 (95% CIs) in %	SICS-105, #*P* = 20 (95% CIs) in %
Accuracy	**89.97** (86.69–93.46)	85.56 (80.63–92.09)	80.87 (75.73–88.97)
Edit	84.33 (77.27–92.61)	**84.52** (75.67–94.72)	78.27 (71.68–86.90)
F1-score	**84.19** (79.67–89.86)	83.04 (76.68–90.51)	75.02 (68.62–84.48)
Sensitivity	**89.97** (86.73–93.46)	85.56 (80.63–92.09)	80.87 (75.73–88.97)
Specificity	97.49 (96.72–98.42)	97.56 (96.52–98.69)	**97.81** (97.19–98.80)
Mean ROC AUC	**99.10** (98.34–99.51)	98.26 (97.16–99.41)	98.32 (97.63–99.34)
Mean PR AUC	**91.14** (90.65–95.56)	89.69 (84.73–94.40)	84.46 (79.80–91.37)

Combining phases as described earlier narrows the performance gap on the SICS-105 dataset compared with the Cataract-101 collection and regarding the edit distance we achieve better results (84.33 vs. 84.52%).

Performance on the Cataract-101 compared to the SICS dataset is higher across most metrics with an accuracy of 89.97 versus 85.56%, an edit score of 84.33 vs. 84.52% and ROC-AUC score 99.10 vs. 98.26%. Specificity and sensitivity on both datasets are comparable (97.49 vs. 97.56% and 89.97 vs. 85.56%). 95%-confidence intervals are narrow and overlap across metrics (see Table 3).

**Fig. 3:**
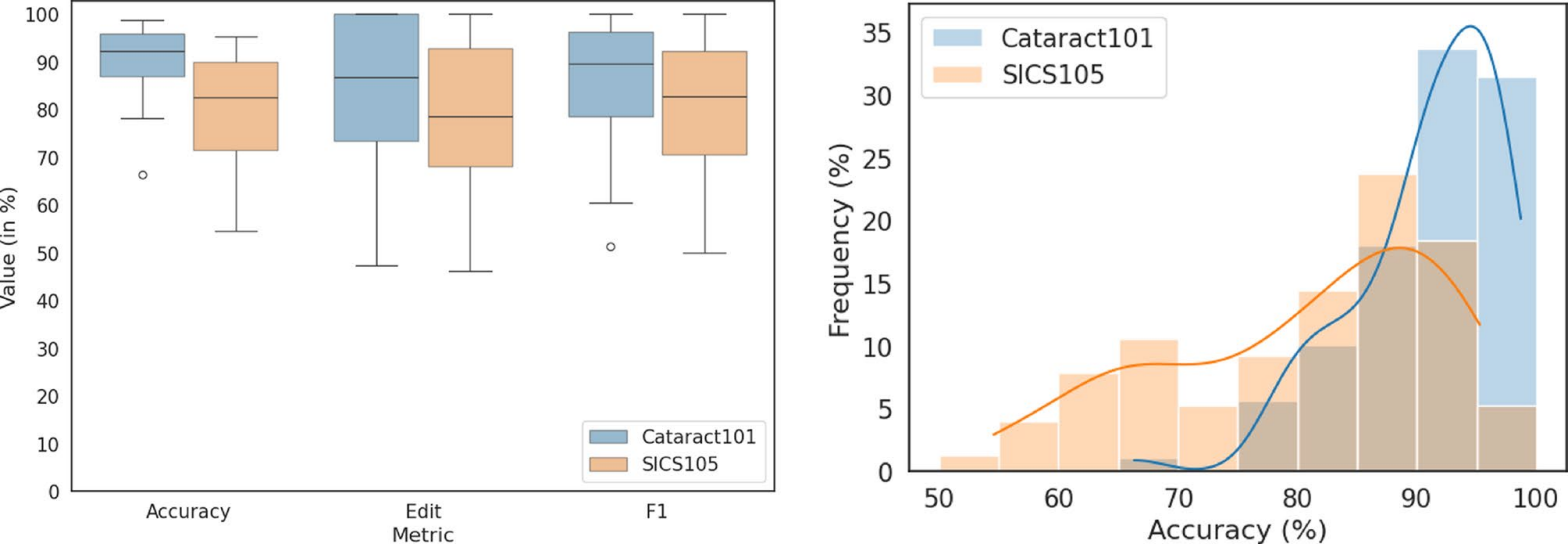
Boxplot (left) and histogram (right) of performance metrics accuracy, edit and F1-score across the validation set of the Cataract-101 (in blue) and SICS-105 (in orange) dataset. The orange and blue lines show an estimated accuracy distribution of the datasets (kernel density function).

Looking at the per-video accuracy distribution in the validation datasets, there seems to be a significant overlap between the two datasets as seen in Fig. 3 (right). The boxplot in the same Figure (left) also shows more performance outliers across all metrics for the SICS data.

The PR-AUC values across phases range from 45.20 to 93.18%. PR curve results for the Cataract-101 videos are consistently higher and vary less (from 68.12 to 97.36% PR-AUC), this can be observed in Supplement Figs. 2 and 3. Note: Supplement Figs. 4 and 5 compare ROC curves for both datasets; they are not discussed here due to near optimal scores.

In another investigation (Supplement Fig. 6), we look at the relationship between video length and edit score. There is a statistically significant, negative correlation between video length and prediction performance with Spearman correlation-coefficient of -0.396 and *p* < 0.01 in the SICS-105 dataset. The same investigation on the Cataract-101 dataset yields a similar, significant Spearman correlation of -0.681 (*p* < 0.01).

### Computational effort

Extraction of i3D features took 20 h for the Cataract-101 and 26 h for the SICS-105 dataset on one NVIDIA TITAN X (Pascal) with 12 GB vRAM. We trained the MS-TCN + + network for 1 h and 57 min (Cataract-101) and 2 h and 2 min (SICS-105).

## Discussion

So far, applications of DL models on cataract surgery videos were limited to phacoemulsification. This research aimed to determine if a DL model (MS-TCN++) could recognize and segment surgical phases in a novel SICS dataset - a technique widely used in LMICs - with comparable performance to its application on the established Cataract-101 phacoemulsification dataset.

Looking at the metrics for the performance of phase recognition, it is clear that phases can be recognized in both phacoemulsification (Cataract-101, IEEE-CATARACTS) and SICS with a high accuracy. The recognition of phases in SICS seems to be slightly more difficult than in phacoemulsification surgery (with an accuracy of 85.56 vs. 89.97%, see Table 3). Comparing our recognition metrics from SICS with other researchers investigating phacoemulsification, we achieve competitive results (see Table 2). The consistently high performance of the modified MS-TCN + + architecture across three different cataract datasets (see Supplement Table 1) proves the generalizability and adaptability of the model to various recording devices and local differences in procedure when properly trained.

The slightly lower performance of our model in SICS may be attributed to the larger number of phases and the longer average surgery duration in SICS compared to phacoemulsification (20 versus 10 phases, 12.95 vs. 8.33 min). Another explanation for the somewhat reduced performance is illustrated by the histogram in Fig. 3 which clusters the accuracy result per video in the validation set in bins. Many videos perform well but there is a tail of cases that the algorithm struggles with. Video length and length variance (see Sect. Materials and methods) are higher in our SICS-105 dataset, which could be explained by higher complexity of some of the included surgeries, where the Cataract-101 collection has more straight-forward cases.

Further analysis of the confusion matrix (see Supplement Fig. 1) for the per-phase prediction accuracy shows that errors mostly occur in the pre- and succeeding phases. This highlights the strength of our model in correctly identifying temporal and spatial patterns of the data. It is very rare that a completely implausible phase is predicted. A part of these “overlap” errors could be explained by the combination of micro-steps in the surgery and the inherent difficulty in finding a discrete timestep where one phase ends and another starts, a problem that is also observed by doctors we trained for the annotation effort and described in the literature^51^.

Also, there is a statistically significant, negative correlation between video length and prediction performance as described in the previous section (see Supplement Fig. 6). This could hint towards a limitation of our DL architecture or the fact that longer videos represent more complicated cases of surgery. These cases often differ in the sequence or length of phases and can contain steps that are not always present in simpler cases leading to an overall underrepresentation. Furthermore, some surgical phases can be repeated in complicated cases, which can create further heterogeneity. The MS-TCN + + publication reports that longer videos present a challenge for the architecture^40^. With an average length of 8:20 min the phacoemulsification videos are shorter than the SICS videos (average length of 12:57 min) which could explain a part of the decreased performance.

As previously described, performance does fluctuate between phases (see Supplement Fig. 2). For example, the phase “sideport” only achieves a PR-AUC of 45.20%. We theorize that this is due to the shortness of the phase (less than 1% of all annotations) and therefore limited amount of training segments. One solution would be to increase the training data size, which would provide more examples to discern similar looking activities.

### Limitations and future perspectives

This study faces limitations regarding the validation of our model. We were not able to validate our MS-TCN + + model on an external dataset because currently there are no other SICS datasets available. Therefore, we can only generalize our results on SICS with limited confidence according to the TRIPOD statement^52^.

From a practical perspective, we expect that more modern frame-wise video features like CLIP or Dino-v2 potentially could increase performance in future studies as demonstrated by some authors and should be investigated^53–55^.

Other potential limitations are variations of surgical protocol within SICS, e.g. with different incision strategies and delivery techniques of the lens nucleus. By raising our dataset from a hospital where a consistent surgical protocol was followed, we only observe the local practices and preferences there^17^. To get a complete picture it would be sensible to collect SICS video recordings from a variety of surgical techniques and from multiple hospitals in different countries, to assess the impact on model performance. This limitation is shared by the Cataract-101 dataset and could be addressed in practice by standardization or fine-tuning a phase network on a selection of training videos including varieties of surgical techniques from different hospitals.

Additionally, it would be interesting to use smartphone-based video recordings in HD of SICS utilizing a beam-splitter to evaluate the algorithm on a low-cost alternative compared to the conventional proprietary recording equipment attached to the microscope. Such a low-cost approach could be reproduced and scaled up much easier in other LMICs^17,26,56^. Finally, the videos in the current dataset have been compressed by a feature of the recording equipment resulting in some blurriness. This is counteracted by downsampling the videos to the same resolution as the Cataract-101 dataset, to increase comparability in the data preprocessing.

The current system could enhance surgeon training by automated retrospective identification of surgical protocol deviations. Future developments may include integrating instrument tracking and complication detection to warn surgeons live during critical phases in the surgery. To implement these improvements, computational limitations need to be addressed, particularly in feature extraction speed (video-inference is quick with an average of 2.43 s, but feature extraction takes up to 20 min) and larger datasets with more complicated examples are necessary to improve the model’s accuracy^53,57^. While this study focuses on SICS, the technical approach has also potential applications in other surgical fields.

Automated surgical phase recognition is an important step towards fully automated analysis of surgical quality as determined by standards like Sim-OSSCAR^32^. Generation of algorithm for automated identification of critical phases, tracking of instruments, and complication recognition are warranted^18,30,31^.

## Conclusion

We conducted the first automated phase segmentation study on Small-Incision Cataract Surgery (SICS) using state-of-the-art artificial intelligence solutions with promising results. With an accuracy of 85.56% and an Area under the Receiver Operator Characteristic Curve (ROC-AUC) of 98.26% we predict the correct phase for most steps in SICS surgery recordings but perform slightly worse than the same algorithm on the Cataract-101 dataset (accuracy of 89.97% and ROC-AUC of 99.10%). Our results on the Cataract-101 dataset with the MS-TCN + + architecture achieve competitive results across metrics with all previous studies done by other research teams.

The observed difference in performance can likely be decreased by larger datasets and using more powerful DL models in the future. In conclusion, this is a first step in automatic assessment of SICS procedures and could be used to improve teaching, reduce its cost in LMICs, automate the recognition of complications and, hence, make this common, but understudied technique safer and more efficient for patients and physicians in the future. We publish our SICS-105 dataset to allow comparison and increase reproducibility for future studies.

## Electronic supplementary material

Below is the link to the electronic supplementary material.

Supplementary Material 1

## Data Availability

The SICS-105 dataset generated during and analyzed during the current study is available in the Zenodo repository, under the following URL https://doi.org/10.5281/zenodo.13847781. The dataset contains surgical phase ground truth, anonymized SICS videos, extracted i3D features, train/validation/test splits and mappings used by the deep neural network. Any additional data can be requested from the corresponding author on reasonable request.Relevant hyperparameters are included in this published article (and its Supplementary Information files). Source code for training and validation of the algorithm are hosted on GitHub under the following URL: https://github.com/AgenoDrei/MS-TCN2-med/.
