# Supplementary material for: Phase recognition in manual Small-Incision cataract surgery with MS-TCN + + on the novel SICS-105 dataset
Source: Sci Rep. 2025 May 21;15:16886. doi: 10.1038/s41598-025-00303-z (PMC12095642; doi:10.1038/s41598-025-00303-z)

# 8 Supplement

## 8.1 Additional external validation with IEEE dataset

Here we supply phase prediction results from another external dataset (as described below) and how we compare them to other authors. This comparison is made difficult by the fact that most prior publications only report very few metrics (mostly accuracy).

**IEEE CATARACTS**: The dataset comprises 50 cataract surgery videos from Brest University Hospital with 18 phases. Patients averaged 61 years old. Microscope videos were recorded with a resolution of 1920x1080 pixels at 30 frames per second. These videos averaged 10 minutes 56 seconds in duration, totaling over nine hours of surgery. Train, validation and test splits are provided and used by this work.

| **Author** | **#P** | **Accuracy** | **ROC AUC** | **PR AUC** | **Sensitivity** | **Specificity** |
| --- | --- | --- | --- | --- | --- | --- |
| This study | 18 | 0.799 | 0.962 | 0.831 | 0.799 | 0.963 |
| **Touma, 2022** (46) | 18 | 0.93 | NR | NR | 0.611 | 0.962 |
| **Lecuyer, 2020** (57) | 18 | 0.623 | NR | NR | NR | NR |
| **Zisimopoulos, 2018** (58) | 18 | 0.783 | NR | NR | NR | NR |

**Supplement Table 1**: Comparing our baselines results on the cataract-101 dataset with phase recognition approaches by other authors in the last 10 years. #P = number of phases used for segmentation. NR = Not reported by the author.

## 8.2 Dataset details

**Cataract-101**: The Cataract-101 collection has 10 distinct surgical phases, which are described by the author as the following (18):

- Incision
- Viscous Agent Injection
- Rhexis
- Hydrodissection
- Phacoemulsification
- Irrigation/Aspiration,
- Capsule Polishing
- Lens Implant
- Viscous Agent Removal
- Tonifying and Antibiotics

**Novel SICS-105**: The following 20 phases are the were initially decided on and annotated with by the ophthalmologists at the Sankara Eye Hospitals:

- Peritomy
- Cautery
- Scleral Groove
- Incision
- Tunnel
- Sideport
- Antibiotics Injection And Wash
- OVD Injection
- Capsulorrhexis
- Main Incision Entry
- Hydroprocedure
- Nucleus Prolapse
- Nucleus Delivery
- Cortical Wash
- Lens Insertion
- OVD Wash
- Stromal Hydration
- Tunnel Suture
- Conjunctival Cautery
- Sup. Rectus Bridle Suture

We combined some of these classes to create a dataset with 13 phases where annotations have a higher similarity with the Cataract-101 collection:

- Conjunctival preparation: Peritomy + Cautery
- Scleral tunnel creation: Scleral groove + Incision + Tunnel:
- Nucleus Removal: Hydroprocedure + Nucleus prolapse + Nucleus delivery
- Wound Closure: Stromal hydration + Tunnel suture + Conjunctival cautery

##

## 8.3 Additional Figures


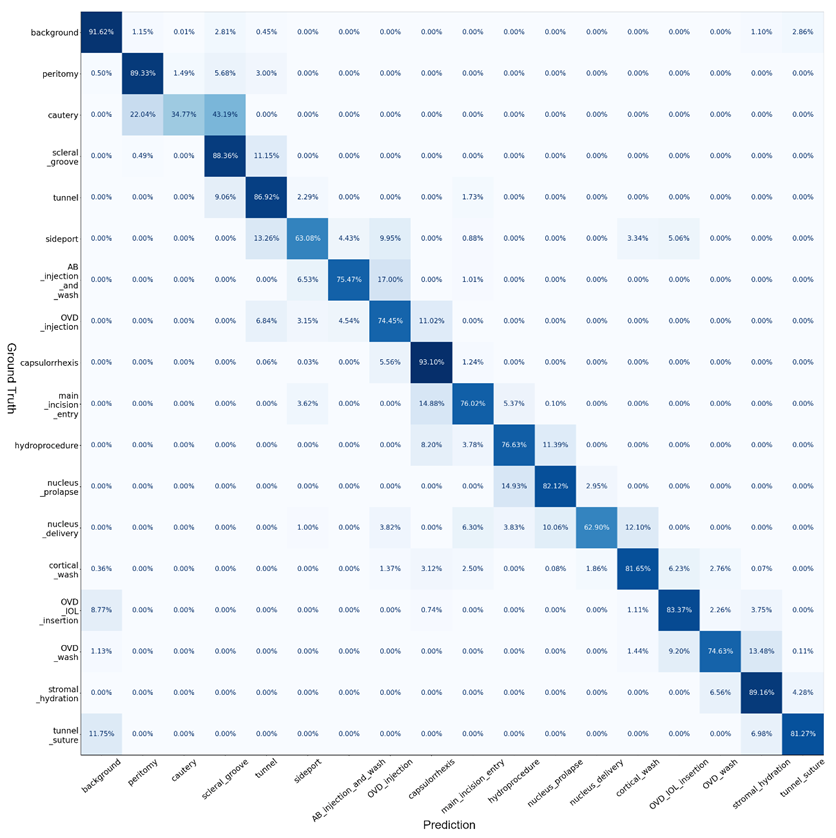


**Supplement Fig. 1**: Confusion matrix (multiclass two-by-two table) between predicted phases and real phases across all validation surgery videos in the SICS dataset. Lighter colors indicate a small number of predicted frames in this phase, and darker colors indicate a high occurrence. The diagonal represents the frames where the prediction matches the ground truth.


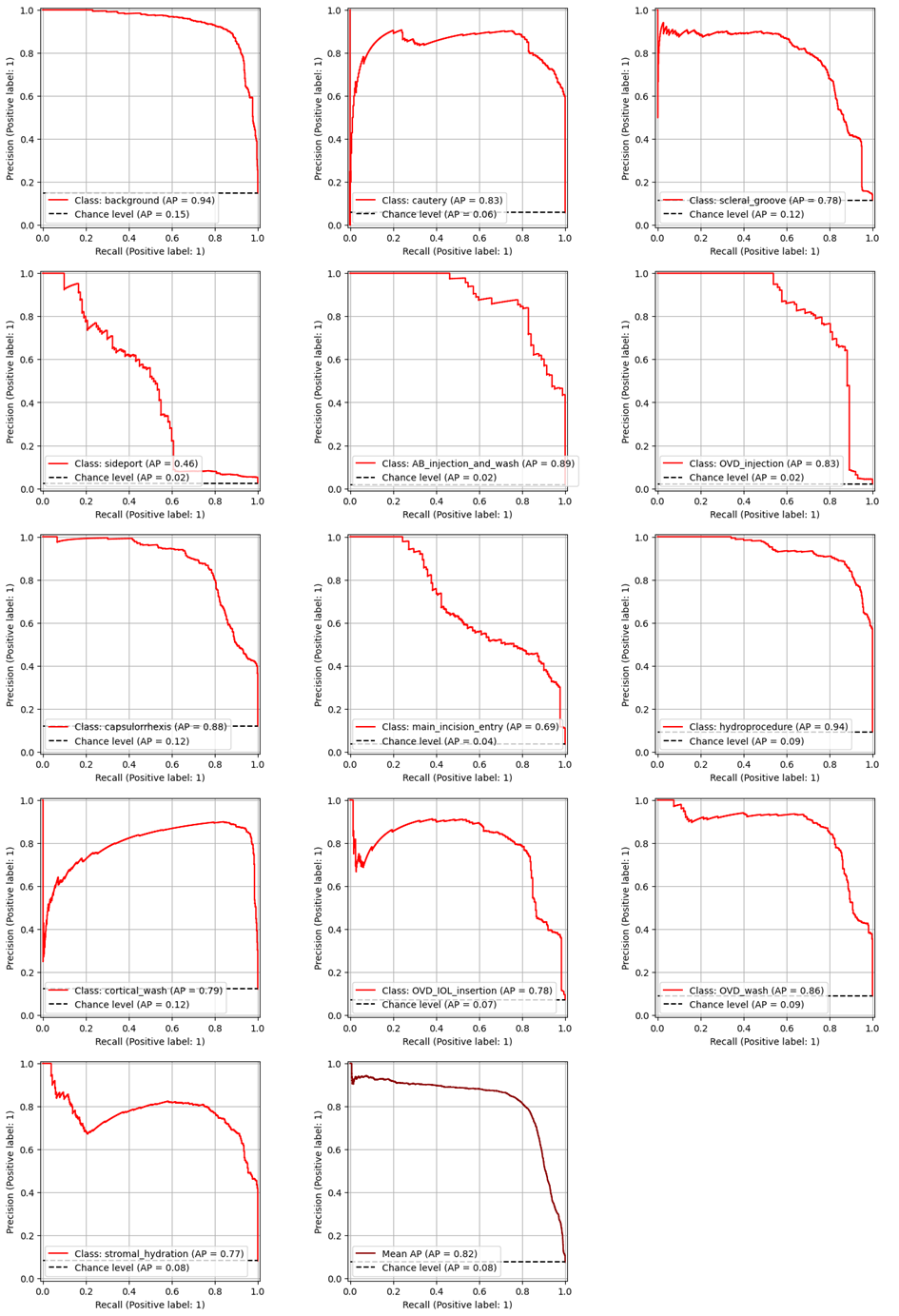


**Supplement Fig. 2**: Precision-recall curves for each phase in the SICS dataset with 13 phases. The last curve represents the average curve for all previous subgraphs.


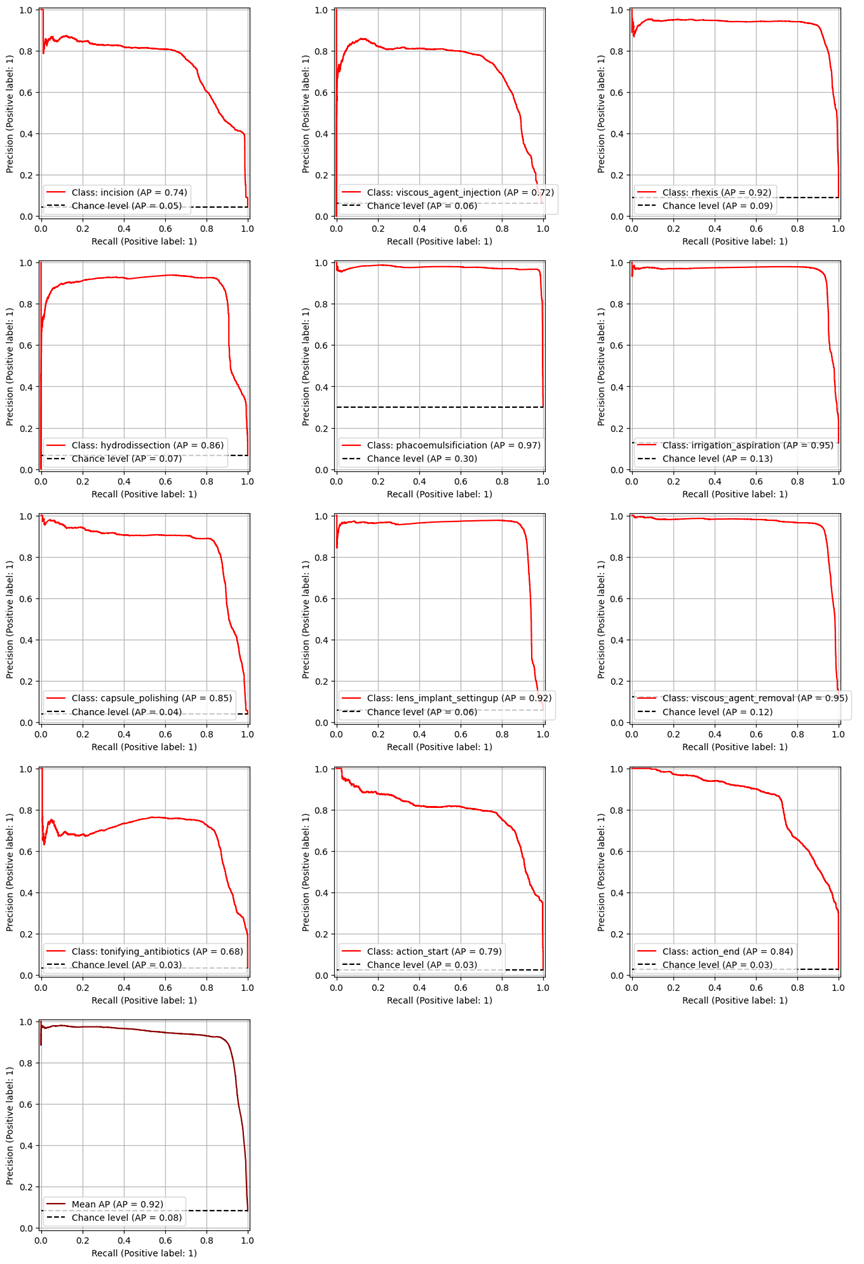


**Supplement Fig. 3**: Precision-recall curves for each class in the Cataract-101 dataset with 10 classes and 2 classes for non-annotated areas in the dataset. The last curve represents the average curve for all previous classes.


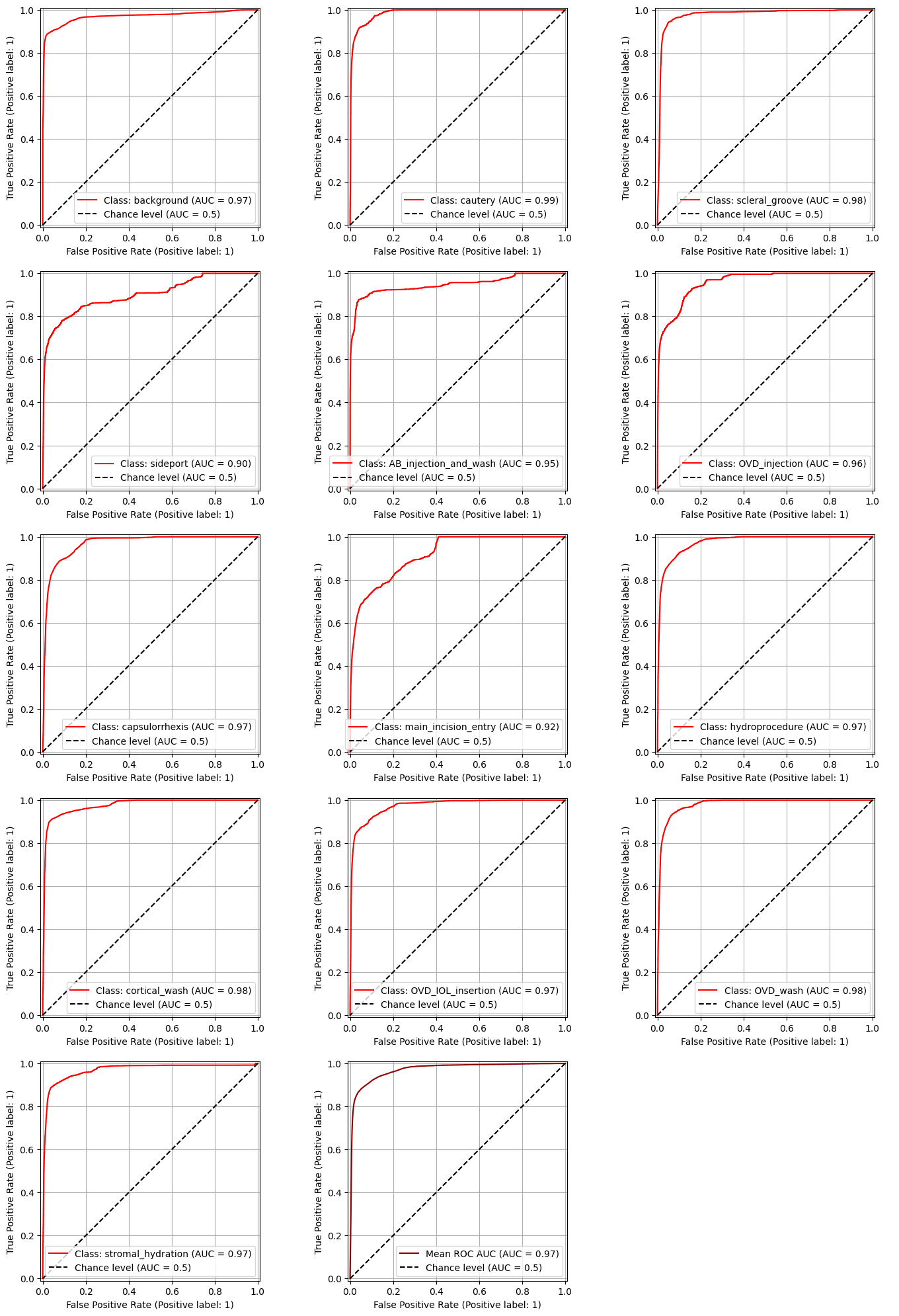


**Supplement Fig. 4**: ROC curves for each class in the SICS dataset with 13 classes and a background class for non-annotated areas in the dataset. The last curve represents the average curve for all previous classes.


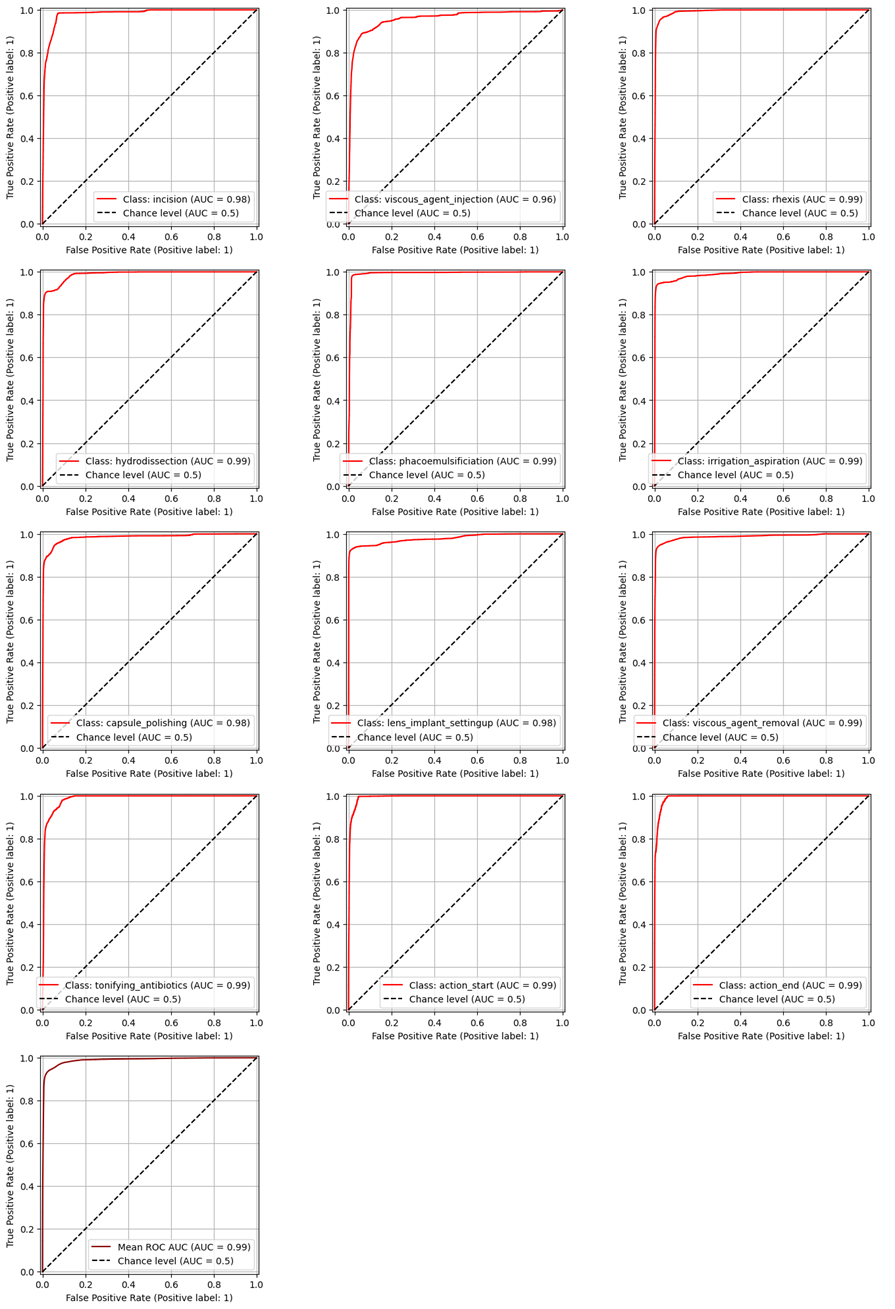


**Supplement Fig. 5**: ROC curves for each class in the Cataract-101 dataset with 10 classes and 2 classes for non-annotated areas in the dataset. The last curve represents the average curve for all previous classes.


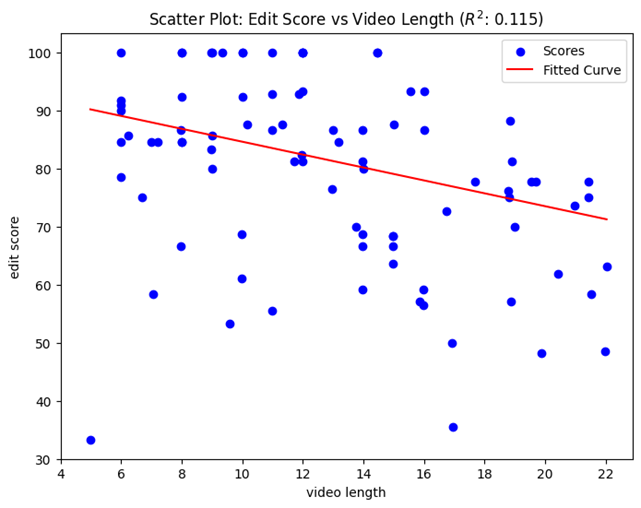


**Supplement Fig. 6**: Scatter plot with video length on the x-axis and the edit-distance score on the y-axis. Each blue dot represents a video in the validation dataset and the red line is a fitted regression line.

## 8.4 Qualitative results

Examples of successful phase prediction of SICS-105:

Example 1:


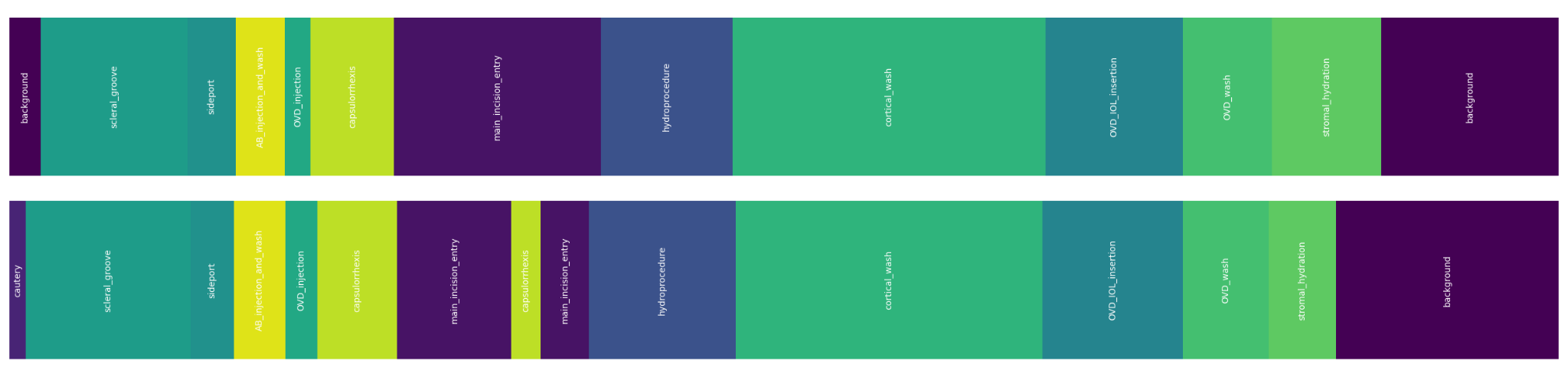


Example 2:


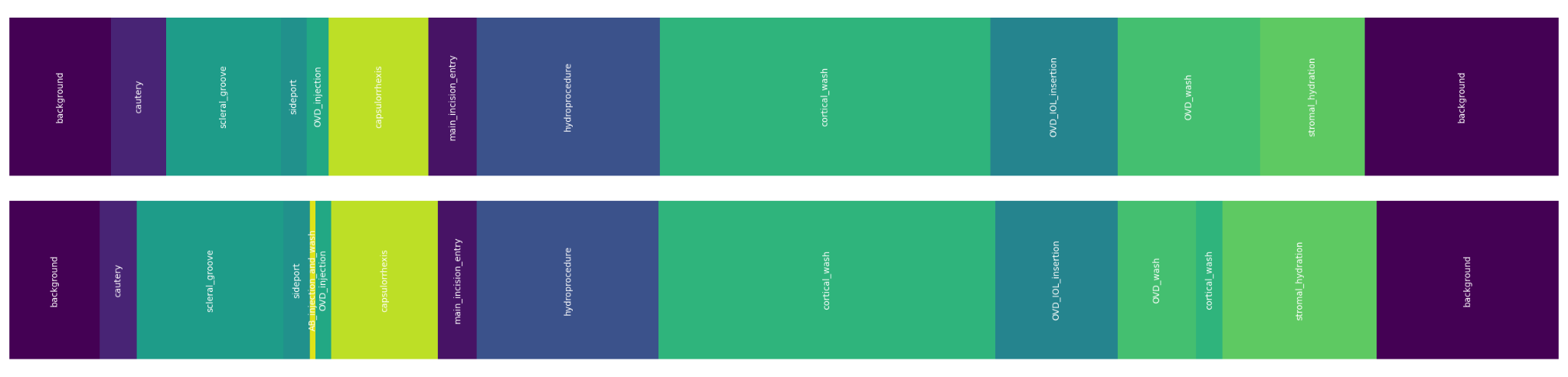


Example 3:


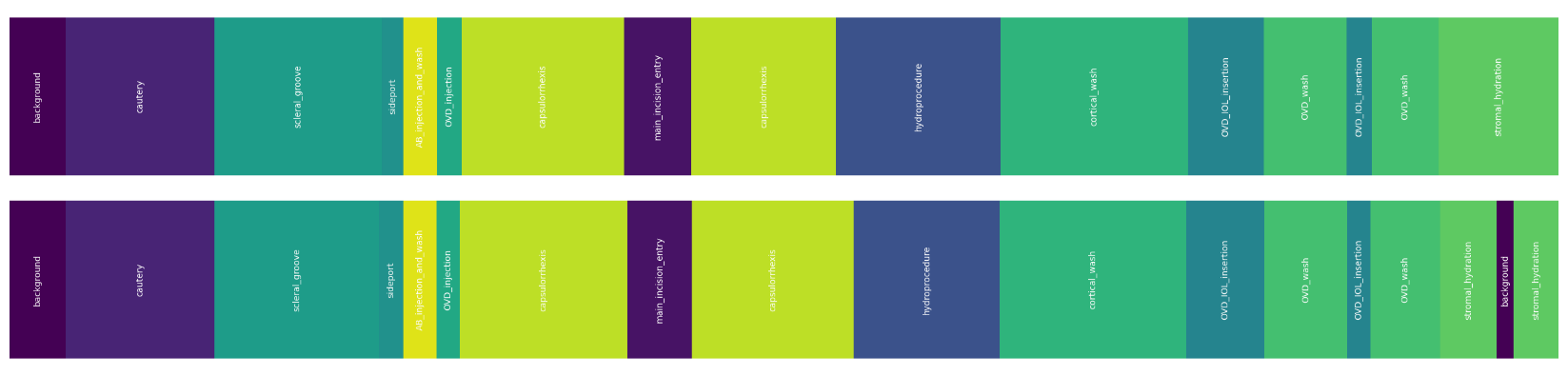


Examples with errors in phases predictions of SICS-105:

Example 4:


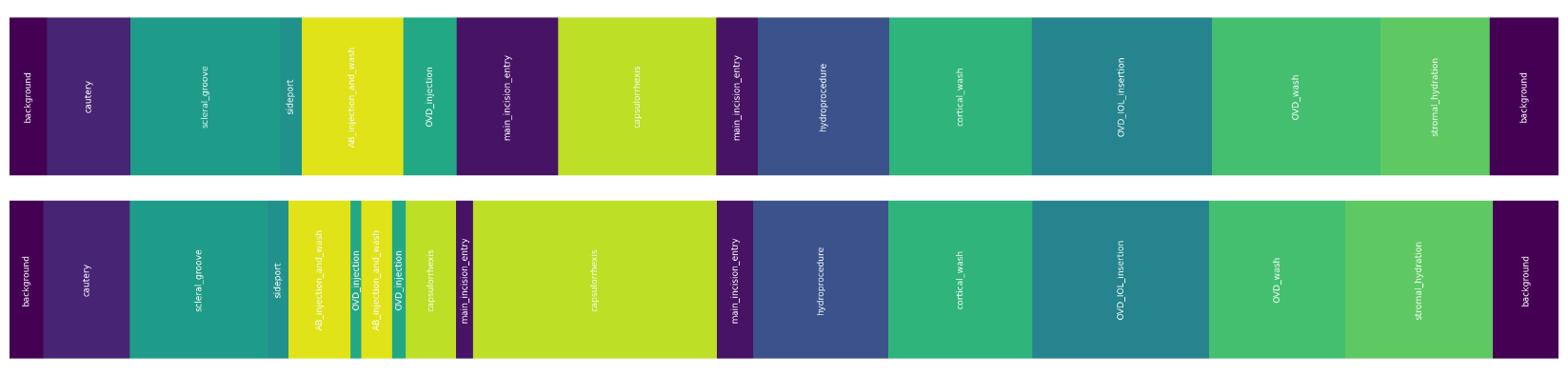

Example 5:


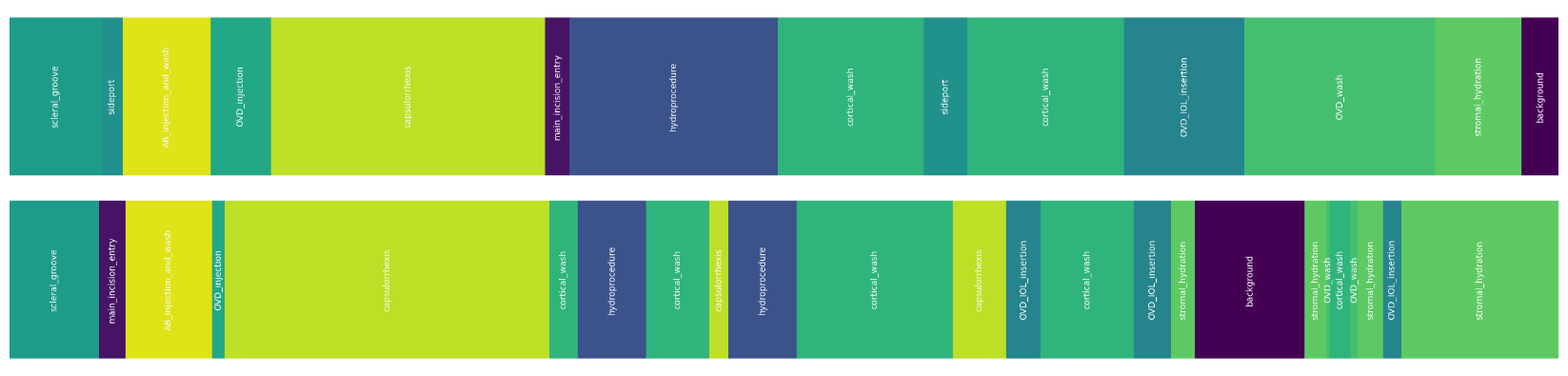


Example 6:


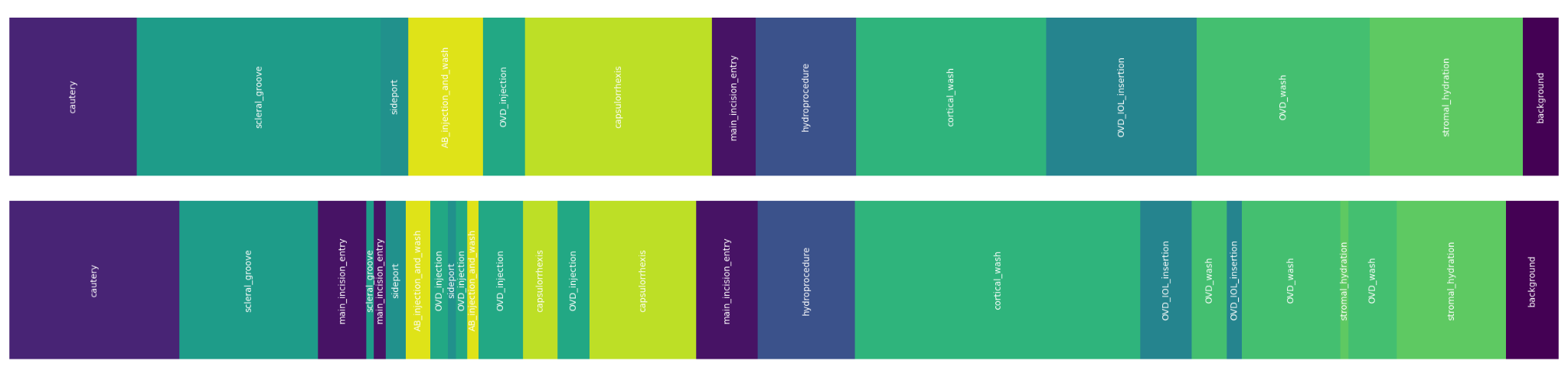

Supplement: Supplementary file 1 — Supplementary Material 1 [file 41598_2025_303_MOESM1_ESM.docx]
